# Incidence and risk factors predisposing anastomotic leak after transhiatal esophagectomy

**DOI:** 10.4103/1817-1737.56012

**Published:** 2009

**Authors:** Abbas Tabatabai, Mozaffar Hashemi, Gholamreza Mohajeri, Mojtaba Ahmadinejad, Ishfaq Abass Khan, Saeid Haghdani

**Affiliations:** *Department of Surgery, Alzahra University Hospital, Isfahan University of Medical Sciences, Isfahan, Iran*; 1 *Department of Shohada Hospital, Lorestan University of Medical Science, Khorram Abad, Iran*; 2 *Department of School of Medicine, Isfahan University of Medical Sciences, Isfahan, Iran*

**Keywords:** Anastomotic leak, risk factor, transhiatal esophagectomy

## Abstract

**OBJECTIVE:**

The objective of our study was to identify the incidence and risk factors of anastomotic leaks following transhiatal esophagectomy (THE).

**MATERIALS AND METHODS:**

A prospective study was conducted on 61 patients treated for carcinoma of the esophagus between 2006 and 2007. We examined the following variables: age, gender, preoperative cardiovascular function, intraoperative complications such as hypotension, arrhythmia, mediastinal manipulation period, blood loss volume, blood transfusion, duration of surgery, postoperative complications such as anastomotic leak, anastomotic stricture, requiring reoperation, respiratory complications, and total morbidity and mortality. Variables were compared between the patients with and without anastomotic leak. T-test for quantitative variables and Chi-square test for qualitative variables were used to find out any relationship. *P* value less than 0.05 was considered significant.

**RESULTS:**

Out of 61 patients, anastomotic leaks occurred in 13 (21.3%). Weight loss, forced expiratory volume (FEV1) <2 lit, preoperative albumin, intaoperative blood loss volume, and respiratory complication were associated with the anastomotic leak in patients undergoing THE. Anastomotic leaks were the leading cause of postoperative morbidity, anastomotic stricture, and reoperation.

**CONCLUSION:**

Anastomotic leakage is a life-threatening postoperative complication. Careful attention to the factors contributing to the development of a leak can reduce the incidence of anastomotic complications postoperatively.

Esophageal anastomotic leak continues to be a significant cause of morbidity and mortality after esophagectomy. The most important predisposing factors are attributed to ischemia of the gastric conduit and errors in surgical technique.[1] The best method of performing an esophagogastric anastomosis remains to be established.[2,3] Cervical anastomoses have been associated with leakage and stricture rates as high as 40% and 50%, respectively, but leak-related mortality is 5% or less.[4] In contrast, reported leakage and stricture rates for thoracic anastomoses are up to 7% and 14%, respectively, but leak-related mortality can be as high as 60%.[5]

The type of anastomosis, esophageal substitute, location of anastomosis (cervical or intrathoracic), whether it is a single or double layer, or done manually or with stapling, the organ used in the anastomoses, the stage of the tumor, the distance from the anastomoses line to the tumoral tissue, additional radiotherapy or chemotherapy used in the treatment, the blood levels of hemoglobin and albumin have been all implicated in the etiology of anastomotic leak.[1,6–11] It has been reported that the most important factors are vascularization, the gastric submucosal tissue oxygen tension, and submucosal collaterall circulation.[1,6,7,10,12,13] The purpose of the present study was to identify predisposing factors and incidence of anastomotic leakage after transhiatal esophagectomy.

## Materials and Methods

Seventy six consecutive patients with carcinoma of esophagus undergone transhiatal esophagectomy (THE) were enrolled in this prospective study between November 2006–2007. All the patients were matched considering some predisposing factors such as anastomosis substitute, route of transposition, anastomosis technique, location of the anastomosis, and lack of chemoradiotherapy before the procedure. Various factors related to the patients such as perioperative conditions were studied. All the patients selected for the study were stable in terms of cardiac and respiratory functions. Dutex AS3 was used to monitor ECG, blood pressure, oxygen saturation, blood gases during the surgery.

THE involves both abdominal and cervical (neck) incision. The thoracic cavity is not opened. The abdominal component of the procedure involves complete mobilization of the stomach. Lymph nodes around the distal part of the esophagus, the gastric cardia, and the left gastric artery are resected in continuity with the specimen. The intrathoracic part of the esophagus is then dissected away from adjacent thoracic structures by using a blunt technique.

To perform this maneuver, the surgeon opens the diaphragmatic hiatus and mobilizes the esophagus by careful manual dissection up into the thoracic cavity. The cervical component of the operation involves opening the neck and retracting the sternocleidomastoid muscle laterally. The part of the esophagus in the neck is encircled and dissected away from the adjacent trachea. The esophagus is then divided in the neck and passed down through the chest. The upper part of the stomach is then divided and the specimen, which includes the esophagus and the upper part of the stomach, is sent to the pathology laboratory for examination. Gastrointestinal continuity is reestablished by constructing a tube out of the remaining part of the stomach and passing the tube up through the posterior mediastinum and anastomosing the cervical part of the esophagus to the stomach tube by hand-sewn, single layer, interrupted suture using 2-0 vicryl. All the operations were done by one surgeon.

The patients were transferred to ICU and were monitored carefully. We kept the thoracic or the cervical drain until the seventh postoperative day in all patients. On the seventh day, we made the patient drink 250 ml of methylene blue and observed whether the colored liquids came from the drains or not. Patients with leakage were observed and fed via jejunostomy tube. Information was collected and variables such as history of smoking, pathology, weight loss, FEV1, location of tumor, serum albumin, intraoperative complications such as hypotension, arrhythmia, mediastinal manipulation period, blood loss volume, blood transfusion, and duration of surgery, postoperative complications such as anastomotic stricture, requiring reoperation, respiratory complications, and total morbidity and mortality were compared between patients with and without anastomotic leak.

Association between qualitative variables with anastomosis leak was assessed using the chi-square statistic or Fisher's exact test. Quantitative variables were assessed by using T-test and *P* value <0.05 was considered statistically significant for all results using SPSS software.

## Results

During the study period, 76 patients were referred for transhiatal esophagestomy for carcinoma of the esophagus at the Alzahra Hospital. Out of the 76 patients, 15 were excluded because of unresectable carcinoma and needing thoracotomy during operation in these patients, and only 61 were included in the study. Mean age of the patients was 61.24 ± 11.48, and 63.04% of them were male.

28.9% of the patients had more than 20% weight loss; the mean loss of weight was 10.42 ± 5.1 kg. Mean serum albumin level before the procedure was 3.13 ±.68 gr/dl. Mean FEV1 was 2.04 ± 0.42 liter; mean procedure duration was 106.31 ± 17.88 mins; mean postoperative bleeding was reported to be 506.55 ±150.68 cc; mean hospitalization period was 12.5 ± 8.11 days; and mean fluid given to patients during operation was 1.58 ± 0.34 liters.

In approximately 50.7% (31/61) of the patients, tumors were detected in the middle one third part of the esophagus. 29.5% of them were cigarette smokers and 62.3% had serum albumin level <3.5 gr/lit that was corrected before the surgery in all of them.

Out of 61 patients, 29 (47.5%) had complications; approximately 34 (55.7%) had pleural rupture, 16 (26.2%) had ventilator complications, 13 (21.3%) had anastomosis leakage, 18 (29.5%) had respiratory complications, and 10 (16.5%) had pleural effusion. The mortality rate was 9.8% in the first 30 days. 28.9% of the patients were reoperated and 13.1% had anastomosis stricture.

In the histopathologic examination, 62.3% were reported to be squamous cell carcinoma (SCC), 36.1% adenocarcinoma, and 1.6% squamous adenocarcinomas.

Finally weight loss, FEV1 less than 2 liters, amount of perioperative blood loss, serum albumin level less than 3.5, and respiratory complications were known to be statistically significant risk factors for the anastomosis leakage [Table 1]. On the other hand, anastomotic leakage leads to the increase of overall complications (*P=* 0.0001), Re-Intubation (*P=* 0.006), stricture of the anastomosis site (*P=* 0.006), reoperation (*P=* 0.001), and mortality of the patients (*P=* 0.07) [Table 2].

**Table 1:** Variables compared between patients with and without anastomotic leak

Variable	Leak		
	
	No (%)	Yes (%)	*P* value
Albumin <3.5	54.3	100	0.002
Hypotension	50	76.9	0.08
Pulmonary complication	20.8	61.5	0.013
Weight loss >20%	17	69.2	0.001
FEV <2 lit	31.3	69.2	0.013
Blood loss (cc)	130.76 ± 486.45	197.41 ± 588.76	0.04
Weight loss (kg)	5.04 ± 9.68	4.4 ± 12.3	0.026
Albumin (g)	0.66 ± 3.20	0.32 ± 2.51	0.0001

**Table 2:** Complications which were statistically significant in patient with anastomotic leak

Variables	Leak		
	
	No (%)	Yes (%)	*P* value
Re-intubation	14.6	53.8	0.006
Stricture	6.3	41.7	0.006
Complications	35.4	92.3	0.0001
Mortality	6.4	23.1	0.07
Reoperation	16.7	69.2	0.001

## Discussion

A number of factors have been indicated as favoring or determining esophageal anastomotic leakage; the type of anastomosis, esophageal substitute, location of anastomosis (cervical or intrathoracic), whether it is a single or double layer, done manually or with stapling, the organ used in the anastomoses, the stage of the tumor, the distance from the anastomoses line to the tumoral tissue, additional radiotherapy or chemotherapy used in the treatment, the blood levels of hemoglobin and serum albumin.[14]

Zieren and coworkers[15] conducted a prospective, random trial of single- versus two-layered closure techniques for cervical gastroesophageal anastomosis, noting a significant higher stricture rate in two-layer closure. Bardini and associates[16] found no difference in outcome between running and interrupted suturing techniques in single-layer cervical anastomosis. Beitler and Urschel[17] pooled results from four randomized comparing stapled and hand-sewn anastomotic technique after esophageal-gastrectomy and found no difference in the incidence of anastomotic leak (stapled 9%, hand-sewn 8%).

Review of the published literature to date reveals that a low anastomotic leak is obtained with either stapled or hand-sewn anastomotic techniques. The preferences and experience of the individual surgeon probably are the most important than the particular method chosen for anastomosis.[14]

Many patients offered esophagectomy for malignant diseases are given neoadjuvent therapy, including chemotherapy and radiations, as part of a multimodality treatment plan. Surgeons have long been concerned that induction therapy would have a negative impact on the mortality and morbidity of the subsequent operations. Most studies to date do not support the hypothesis,[10,18,19] although at least one study found neoadjuvent therapy to be a risk factor for anastomotic leak in multivariate analysis.[20] Higher anastomotic leak rates have been reported in patients undergoing salvage esophagectomy after definitive chemotherapy and radiotherapy.

The esophagus lacks a serosal layer, and the often fragile outer longitudinal muscle layer holds sutures poorly. This relative inability to hold suture may contribute to the higher leak rate occasionally reported with a running-suture technique.[18] The intrathoracic route of the translocated conduit may contribute to the leak rate; the substernal route is associated with a higher rate than seen with placement of the conduit through the bed of the resected esophagus.[15,20] One may theorize that this higher rate is seen because the substernal route is longer and may lead to increased compression of the conduit at the level of the thoracic inlet.

Surprisingly, malignant infiltration of anastomotic tissues is a questionable risk factor for esophagogastric anastomotic leakage. Some investigators have found positive resection margins to be causally related to anastomotic leaks,[10] but others have not.[21,22] An increase in the amount of intraoperative bleeding leads to the increase in anastomotic leakage. In our study, patients with increase in intraoperative bleeding had significant leakage (*P* < 0.05). Presence of diabetes mellitus[18,22] or advanced age[22] seems to have little effect on the development of anastomotic leak.

Akiyama[23] has emphasized the importance of hypoalbuminemia as a risk factor, while Patil and colleagues[10] reported a positive correlation between anastomotic leakage and an albumin level less than 30 g/L. In our study, serum albumin in patients with anastomotic leak was significantly low (*P* = .0001) and patients with serum albumin below 3.5 g/dl had higher anastomotic leak (*P* = .002). Low serum albumin indicates malnutrition; in our study, we made it clear that there is a strong relationship between serum albumin and anastomotic leak. So, we recommend correcting nutritional conditions in patients before the procedure by tube jejenostomy rather than parental nutrition.

Patients with FEV1 less than 2 liters had higher anastomotic leak (*P* = .013) which may be due to the role of hypoxia on conduit ischemia which is the most important factor known for the anastomotic leak. Patients with respiratory and ventilatory complications after the procedure had significant anastomotic leak that shows the negative role of hypoxia on tissue repair.

Incidence of anastomotic leak in our study was 21.3%, which was more compared to other studies.[20] One possible cause is that in these studies, patients selected were randomly comprised of both benign and malignant tumors, but in our study more than 90% of the patients had stage III malignancies. In our study, anastomotic leak lead to increase in complications (*P* = .0001) including stricture of the anastomotic site (*P* = .006). Dewar[18] and the coworkers analyzed the association between intraoperative bleeding, anastomotic leakage and stricture of the anastomotic site (*P* = 0.04) that was same as our results. Presently, no effect was on the mortality rate (*P* = .07).

In summary, the incidence of esophageal anastomosis leakage was 21.3% with no effect on mortality rate. Risk factors for anastomotic leak are FEV1 less than 2 liters, intraoperative bleeding, low serum albumin, respiratory complications, and loss of weight. Also, anastomotic leakage leads to increase in overall complications, stricture of anastomotic site, re-intubation and reoperation. Finally, we recommend nutritional status of the patients suffering from esophageal cancers should be corrected by tube jejenostomy rather than parental nutrition and the patients with unfavorable respiratory conditions should be treated because respiratory complications are the most common cause of mortality in these patients.[24]
